# Case Report: Diagnostic and management challenges of necrotizing fasciitis in an immunocompromised child with acute lymphoblastic leukaemia

**DOI:** 10.3389/fsurg.2026.1898849

**Published:** 2026-10-22

**Authors:** Meng Xian Chan, Nazurah Jaafar Sidek, Arief Sadiqin Azaharie Mohamed Zahari, Azrina Syarizad Kuthubul Zaman, Adzim Poh Yuen Wen, C-Khai Loh, Sie Chong Doris Lau, Marjmin Osman

**Affiliations:** 1 Faculty of Medicine, Universiti Kebangsaan Malaysia, Kuala Lumpur, Malaysia; 2 Pediatric Surgery Unit, Department of Surgery, Faculty of Medicine, Universiti Kebangsaan Malaysia, Kuala Lumpur, Malaysia; 3 Plastic and Reconstructive Unit, Department of Surgery, Faculty of Medicine, Universiti Kebangsaan Malaysia, Kuala Lumpur, Malaysia; 4 Department of Pediatrics, Faculty of Medicine, Universiti Kebangsaan Malaysia, Kuala Lumpur, Malaysia

**Keywords:** Fournier gangrene, leukaemia, negative-pressure wound therapy, paediatric oncology, paediatric surgery, perineum, plastic and reconstruction, skin allograft

## Abstract

Necrotizing fasciitis (NF) is a rare, rapidly progressive soft-tissue infection that, when compounded by an immunocompromised state, increases susceptibility, severity, and diagnostic difficulty. Furthermore, reconstruction of the resulting defects is particularly challenging in neutropenic patients. Skin allograft offers a temporary biological cover that may be especially advantageous in this setting. We describe its use in a child with relapsed Philadelphia chromosome-positive acute lymphoblastic leukaemia (Ph + ALL) who presented with NF of the perineum while on chemotherapy. A 4-year-old boy with relapsed Ph + ALL on a high-risk chemotherapy protocol presented with neutropenic fever and decompensated septic shock. Despite resuscitation and broad-spectrum antibiotics, he deteriorated, requiring intensive care. A perianal swelling initially attributed to cellulitis progressed, and rapid clinical deterioration raised suspicion of perineal NF. He underwent emergency exploration, which revealed extensive non-viable tissue of the perineum, bilateral inguinal regions, and thigh; serial debridement and a diverting colostomy were performed, with tissue cultures yielding *Proteus mirabilis* sensitive to cefepime and meropenem. Reconstruction was staged with negative-pressure wound therapy and human skin allograft. Allograft was preferred over autograft because the child was physiologically unstable with an unhealthy wound bed, and because delayed allograft rejection in the immunosuppressed permits prolonged engraftment. The wound healed over 5 months, and the stoma was reversed at 5 months. Skin allograft served as an effective temporary biological dressing that prepared the wound bed and promoted healing while avoiding the donor-site morbidity of an autograft in a critically ill child, with immunosuppression likely favouring prolonged engraftment. This case adds to the limited evidence supporting allograft as a useful adjunct for staged wound closure in immunocompromised children with NF. NF in immunocompromised children is easily missed and demands a high index of suspicion and prompt surgical control. Skin allograft is a useful, well-tolerated adjunct for staged wound closure in this group, avoiding donor-site morbidity in a critically ill child; larger studies are needed to define its role.

## Introduction

Necrotizing fasciitis (NF) is a rapidly progressive soft-tissue infection characterized by extensive necrosis of the fascia and subcutaneous tissues (1). The incidence in the paediatric age group is relatively rare, with an estimated range of 0.02–0.08 per 100,000 children per year (1). Although NF can occur in otherwise healthy children, certain factors, such as an immunocompromised state, can increase susceptibility and severity of the disease (2).

NF can be divided into Type I and Type II infections (1). Type I infection is polymicrobial and commonly seen in adults. In contrast, Type II infection is monomicrobial (1). NF in association with acute lymphoblastic leukaemia (ALL) has been described in only six case reports (3–8). It usually occurs during the granulocytopenic phase of chemotherapy (8). Due to the underlying disease, managing NF in these children poses a challenge in timely diagnosis and treatment.

Diagnosis is often delayed in this group of children because of the subtle signs and symptoms of immunosuppression. Even the most commonly described clinical signs, which include disproportionate pain, erythema, tenderness, warm and swollen skin, blistering, skin crepitus, and necrosis, are only observed in 10%–40% of all patients (9). The initial presentation is often subtle, involving fever, tachycardia, and skin erythema that resemble neutropenic fever and cellulitis in this group of children.

Laboratory results may also be difficult to interpret due to the blunted immune response in leukaemia, which often presents with neutropenia (1). Laboratory risk indicator for necrotizing fasciitis (LRINEC) scoring systems used in adults to distinguish NF from other soft-tissue infections are not validated in the paediatric age group (10). This makes early detection of NF challenging in immunocompromised children.

We present a case of NF of the perineum in a child with ALL, highlighting the challenges in managing such a patient and the usage of skin allograft in an immunocompromised individual.

## Case report

A timeline summarizing the key clinical events is provided in Table 1. A 4-year-old boy with a relapse of Philadelphia chromosome-positive B-cell ALL (Ph + ALL), on a high-risk chemotherapy protocol, presented with fever, vomiting, and loose stool for 1 day. He was diagnosed with Ph + ALL at 2 years old, with the initial presentation of multiple lymphadenopathy and splenomegaly. He was on the ESPHALL/COG protocol in the high-risk delay intensification phase for chemotherapy at the time of this presentation. The boy presented with low-grade fever associated with vomiting and loose stool for 1 day. On examination, the child was lethargic and in decompensated shock. His weight was 12.7 kg and his height was 96.5 cm, giving a body mass index of 13.64 kg/m^2^ (between the 2nd and 16th centiles).

**Table 1:** Timeline of the patient's clinical course and follow-up.

Date since presentation	Events
Day 1	Presented with neutropenic fever and decompensated septic shock, and was admitted to PICU for resuscitation and inotropic support A small swelling over the perianal region was noted USG showed a small non-liquefied collection over the perianal region measuring 1.0 cm × 0.9 cm × 2.3 cm with surrounding subcutaneous and muscle oedema Diagnosis: perianal cellulitis Started IV cefepime 50 mg/kg TDS, IV amikacin 15 mg/kg OD, and IV metronidazole 7.5 mg/kg TDS. Blood culture and sensitivity: Central and peripheral cultures showed *Pseudomonas aeruginosa* sensitive to cefepime and meropenem
Day 5	Swelling remains the same size. Repeat USG showed the subcutaneous oedema had progressed to the left inguinal area, with generalized subcutaneous oedema over the perianal region, and noted a hypoechoic collection mainly at the 12, 1, and 3 o’clock positions of the perianal region Revised diagnosis: NF of the perineum
Day 6	First wound debridement with left diverting colostomy Discontinued IV cefepime, IV amikacin, and IV Flagyl after 6 days Started IV meropenem 40 mg/kg TDS and IV voriconazole 8 mg/kg BD
Day 7	Started IV teicoplanin 250 mg/m^2^ OD
Day 8	Second wound debridement
Day 11	Third wound debridement + NPWT dressing
Day 15	Patient is extubated and not on inotropic support
Day 18	Reapplication of vacuum dressing Tissue culture and sensitivity: *Proteus mirabilis* sensitive to Unasyn
Day 22	Discontinued IV teicoplanin after 15 days Started IV Unasyn 50 mg/kg
Day 24	Transferred from PICU to ward IV voriconazole changed to oral route 100 mg
Day 27	Discontinued IV meropenem after 22 days
Day 29	Discontinued oral voriconazole after 24 days Started oral itraconazole
Day 32	Reapplication of vacuum dressing
Day 38	Fourth wound debridement Toilet and secondary suturing of left flank wound Reapplication of vacuum dressing
Day 44	Fifth wound debridement Allograft application Reapplication of vacuum dressing
Day 51	Discontinued IV Unasyn after 30 days Started oral Augmentin 350 mg BD until discharge
Day 54	Reapplication of vacuum dressing
Day 59	Patient discharged home Reapplication of vacuum dressing
Day 65–100	Re-admitted for *Candida orthopsilosis* urinary tract infection and non-neutropenic fever Wound inspection and reapplication of vacuum dressing weekly Restarted immunotherapy: blinatumomab Discharged from ward on Day 100
Day 100–162	Wound inspection and dressing every 2 weeks in daycare
Day 162	Perineal wound completely healed
Day 163	Reversal of left transverse colostomy

Laboratory tests revealed bicytopenia, with a total white cell count of 0.14 × 10^9^/L and a platelet count of 24 × 10^9^/L, alongside elevated C-reactive protein (CRP) at 154 mg/L. With no obvious foci of infection, a diagnosis of neutropenic fever with decompensated septic shock was made, and the child was started on fluid resuscitation and intravenous (IV) cefepime 50 mg/kg TDS as broad-spectrum antibiotics. Despite fluid resuscitation and antibiotics, the child did not show improvement. He was then admitted to the paediatric intensive care unit (PICU) and started on inotropic support.

Subsequently, a small swelling over the perianal region was noted (Figure 1). Ultrasonography (USG) was performed, which showed a small non-liquefied collection measuring 1.0 cm × 0.9 cm × 2.3 cm over the perianal region, with surrounding subcutaneous and muscular oedema. The patient was diagnosed with perianal cellulitis, and IV amikacin 15 mg/kg OD and IV metronidazole 7.5 mg/kg TDS were added as antimicrobial therapy.

**Figure 1:**
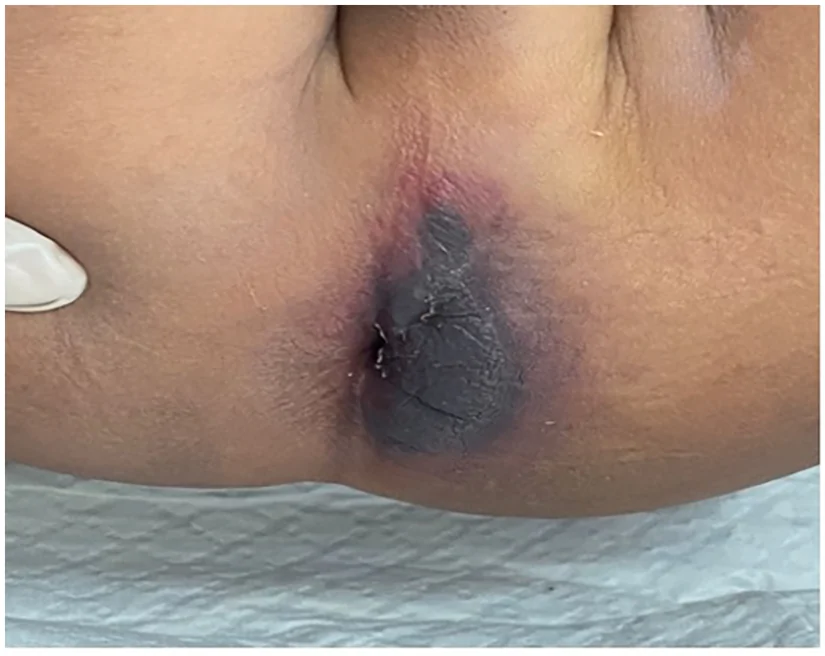
Appearance of the perineum when the child was first referred to paediatric surgery. Discolouration at the perianal region from 12 to 6 o’clock with minimal surrounding erythema.

Unfortunately, the child continued to deteriorate, requiring ventilatory support and increasing inotropic support. He progressed to septic shock with multiorgan involvement, coagulopathy, acute kidney injury, and liver impairment. *Pseudomonas aeruginosa* was isolated from blood culture and was sensitive to cefepime and meropenem. Thus, antimicrobial therapy was escalated to IV meropenem 40 mg/kg TDS, IV voriconazole 8 mg/kg BD, and IV teicoplanin 250 mg/m^2^ OD while the previous antimicrobial therapy was discontinued.

Since the swelling remained unchanged after 5 days of admission, a repeat USG of the groin was performed, which showed that subcutaneous oedema had progressed to the left inguinal area, with generalized subcutaneous oedema over the perianal region. These findings, coupled with rapid clinical deterioration, raised a strong suspicion of NF of the perineum. Thus, he underwent immediate surgical exploration. Intraoperatively, unhealthy tissue was identified in the perianal region, perineal body, bilateral inguinal regions, and left upper medial thigh. Extensive wound debridement was performed, together with a diverting transverse colostomy (Figure 2).

**Figure 2:**
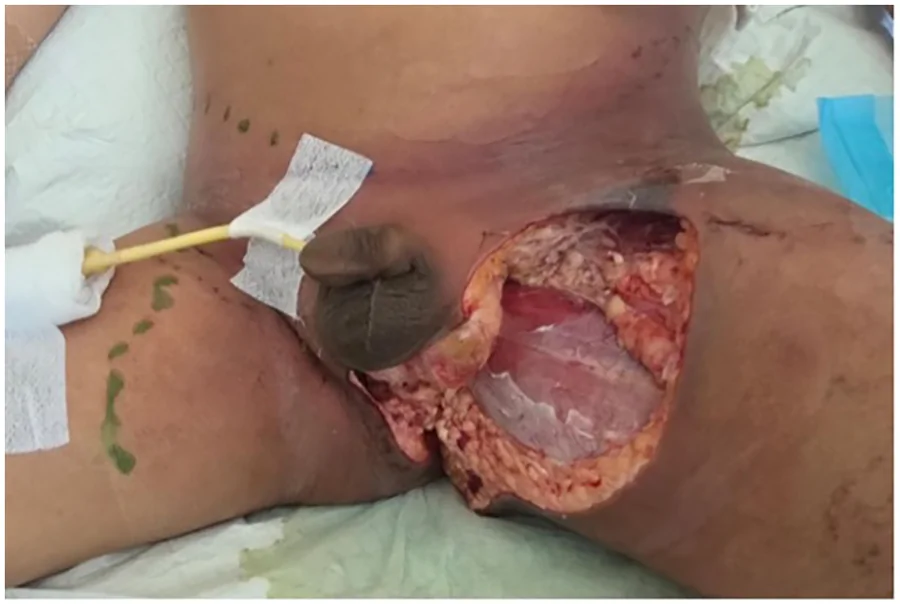
Wound after first wound debridement. Noted erythema over the left lower abdomen and left flank.

The child clinically improved following multiple wound debridements. Blood cultures since the first debridement had shown no growth. Tissue culture of the perineum isolated *Proteus mirabilis* sensitive to Unasyn. Thus, IV teicoplanin was discontinued and switched to IV Unasyn 50 mg/kg. Toilet and secondary suturing of the wound at the left flank was performed on Day 38 of admission. Multiple cycles of negative-pressure wound therapy (NPWT) dressings were applied to aid wound closure (Figure 3). Despite NPWT for 1 month, the wound size did not improve significantly; therefore, the decision for a skin allograft was made.

**Figure 3:**
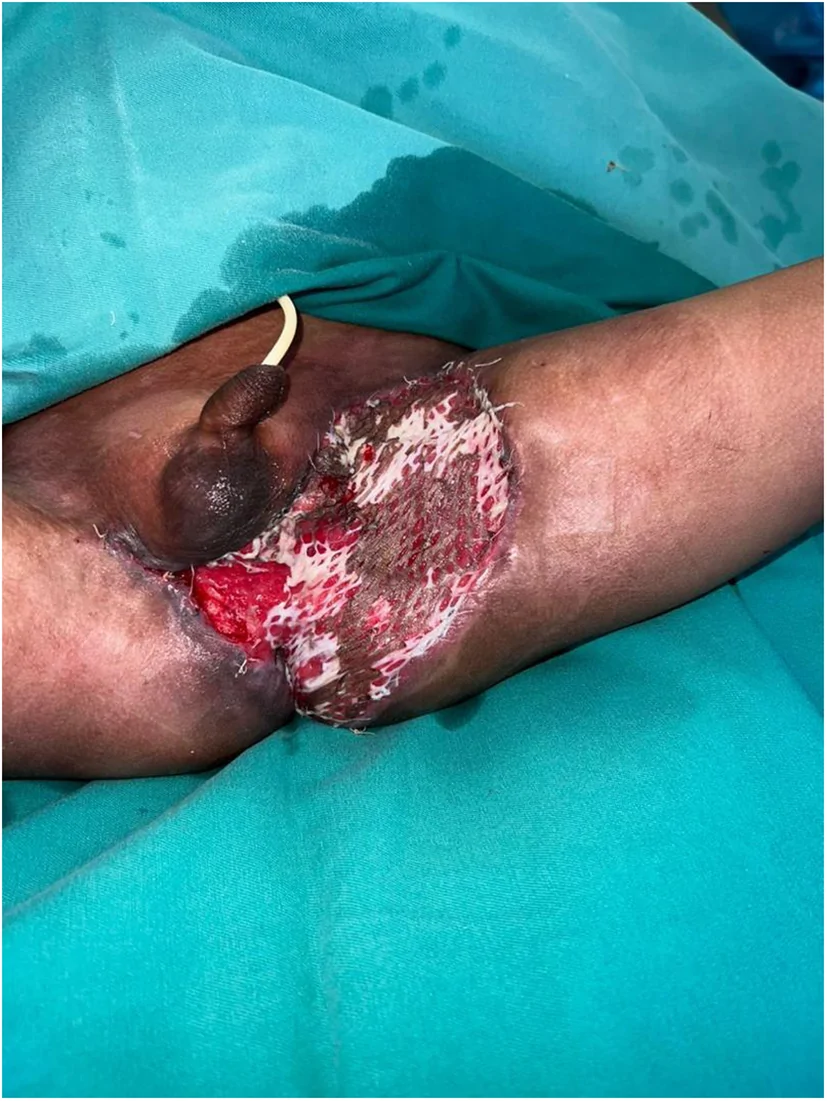
The wound bed appearance 1 week after the application of skin allograft.

The patient underwent application of an Human leukocyte antigen (HLA)-unmatched allograft obtained from our local skin bank on Day 44 of admission. The application of the allograft was aided with NPWT covering it. A weekly inspection of the wound and change of the NPWT were performed. At 1 week after allograft application, there was already 70% allograft uptake, with epidermolysis and whitish dermis observed. Allograft uptake increased to 80% at 2 weeks post-allograft application. The child was eventually discharged home with NPWT dressings after 2 months of admission. It took 5 months after the initial presentation for the wound to heal completely (Figure 4), and the stoma was reversed the next day.

**Figure 4:**
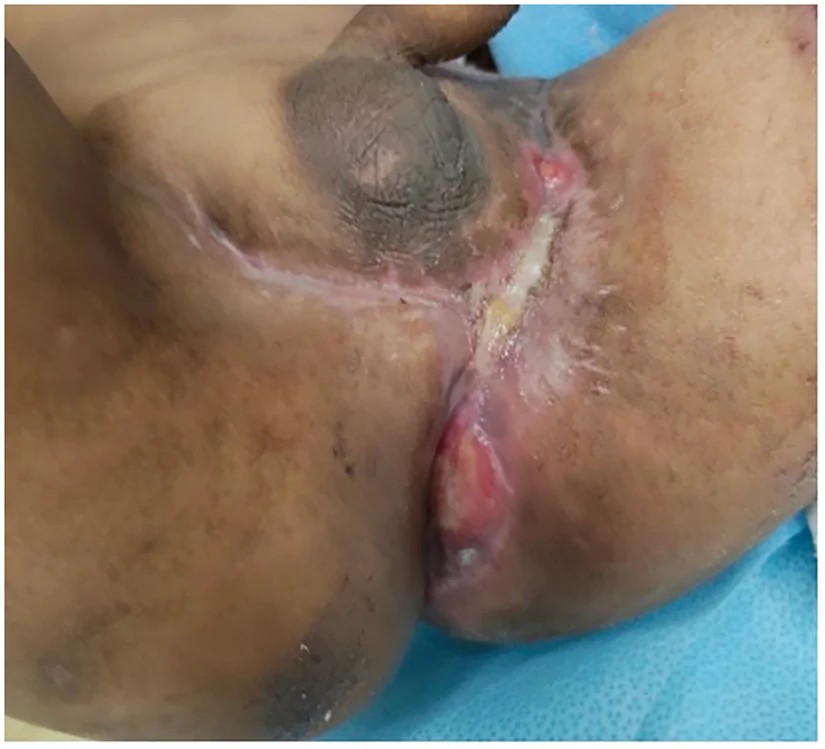
Wound at 4 months following the first debridement.

## Timeline

See Table 1.

## Discussion

Necrotizing fasciitis in immunocompromised patients carries an in-hospital mortality of 39.1% compared to 19.4% in immunocompetent patients (2). Most cases of NF in ALL reported blood culture-positive organisms, which are *Pseudomonas aeruginosa*, consistent with our patient (3, 6, 7). *P. mirabilis*, on the other hand, is an uncommon isolate in necrotizing fasciitis (11) and is commonly found in the gastrointestinal and genitourinary tracts (12). One study postulated that the urinary tract could be the source of *P. mirabilis* for NF (13).

In imaging for diagnosing NF, ultrasonography is a useful modality with a sensitivity of 75%–88.2% and a specificity of 70.2%–93.3% (14, 15). Ultrasound distinguishes a localized abscess from rapidly spreading necrotizing fasciitis by assessing whether the fluid collection is well-defined and encapsulated or diffusely tracks along deep tissue layers. Meanwhile, an abscess appears as a well-defined hypoechoic fluid collection on ultrasound. In our patient, the collection was non-liquefied and the spread was not documented; thus, a perianal cellulitis was initially diagnosed. Although a computed tomography (CT) scan can reveal deep fascial thickening and fluid and gas within soft-tissue planes in and around the superficial fascia, with a sensitivity of 88.5% and specificity of 93.3%, it ranks second as an option due to the radiation exposure (16). Magnetic resonance imaging (MRI) is also equally effective; however, these patients are often ill and difficult to transport to the MRI suite. Nonetheless, none of these imaging modalities should delay definitive surgery and are useful only as adjuncts (1). We suggest that a high index of suspicion should always dictate management despite these findings in immunocompromised patients.

Surgery remains the mainstay of treatment for tissue necrosis (17). Antibiotics alone are ineffective because they cannot reach necrotic tissue. The average mortality of NF in paediatric patients is 15%, and the mortality rate increases significantly when surgery is delayed for more than 6 h (18, 19). Extensive wound debridement is key to improving clinical outcomes and may require several procedures. However, the difficulty lies in distinguishing necrotic tissue from normal healthy tissue. Furthermore, the risk of bleeding outweighs the depth of debridement and is exacerbated in immunocompromised patients, as they tend to bleed easily due to thrombocytopenia and coagulopathy. The principle of “radical excision until healthy bleeding tissue” can be difficult to apply in immunocompromised children, especially when the child is on inotropic support, as viable tissue may appear dusky or non-viable due to poor perfusion rather than necrosis. Serial debridement with reassessment is crucial. The use of NPWT helps to accelerate healing. Another challenge is that the patient will require a balance between surgical excision, which necessitates time for the serial intervals, and speeding up wound closure to allow chemotherapy to proceed on schedule.

A left transverse colostomy was performed to divert faecal material. An open, debrided wound would be placed in contact with stool, which exposes the wound to enteric pathogens. This would increase bacterial load, impair granulation, and dislodge skin grafts. Thus, diversion of faecal material, most commonly via colostomy or non-operatively via a flexible faecal management catheter, was intended as source control of contamination (20).

Wound healing in these patients often takes longer than in healthy children due to neutropenia and an overall immunocompromised state (21). We chose to use allograft because the wound area is too large for primary closure, and the wound size is not decreasing despite repeated NPWT. It is used as a temporary biological dressing as part of staged reconstruction of complex wounds (22). It has also been shown to shorten the time to epithelialization. Allograft works through multiple mechanisms. It reduces evaporative loss of water, electrolytes, heat, and protein from the wound by preventing fluid evaporation (23). It also provides a dermal extracellular matrix scaffold rich in collagen and growth factors, creating a favourable environment for fibroblast migration, angiogenesis, and keratinocyte growth (24). Allograft also suppresses microbial proliferation and bacterial bioburden, thus further enhancing wound improvement (25).

The use of allografts in immunocompromised patients with NF is more beneficial than in immunocompetent patients, as immunosuppressed patients have delayed rejection and may permit prolonged engraftment compared to immunocompetent patients (26). Other options include autografts, myocutaneous flaps, NPWT, and healing by secondary intention. However, any autologous tissues are also at risk of creating risks to a new donor wound in immunocompromised patients, including poor integration and graft failure. NPWT seems to work synergistically with allograft by increasing the rate of allograft uptake, as after 8 days of NPWT there was already 70% uptake. Across all case reports of NF in association with ALL, none used an allograft for wound closure, underscoring the novelty of our case. Skin allograft was often used as temporary coverage for extensive burn wounds (27), and a recent study shows they can accelerate wound healing in diabetic foot ulcers (28).

While it is critical to treat the acute NF, one must not lose sight of the underlying leukaemia. Ongoing coordination with the oncology team is essential to determine the optimal timing for the resumption of chemotherapy and steroids, carefully weighing the risks and benefits.

## Limitation

As a single case report, this paper carries the inherent constraints of its design. It can illustrate management principles and generate hypotheses, but it cannot establish causation, define incidence, or support generalizable treatment recommendations. Because the child was managed with several concurrent interventions—a diverting colostomy, negative-pressure wound therapy, skin allograft, and carefully timed resumption of chemotherapy—the independent contribution of any one of these to the eventual healing cannot be isolated. These findings may not extend to other organisms, anatomical sites, or immunocompromised states.

## Conclusion

Managing NF in immunocompromised children with leukaemia is particularly challenging due to rapid disease progression, subtle clinical signs, and diagnostic difficulties. This case shows that NF must be suspected when an immunocompromised child presents with skin swelling and discolouration, as misdiagnosis allows NF to spread rapidly to nearby tissue. Their weakened immunity complicates management, slows wound healing, and increases susceptibility to secondary infections. Allograft application supplemented with NPWT shows promising outcomes in secondary wound closure for our patient. The application of allograft offers a new possibility in the wound management of immunocompromised children. Further clinical studies involving a larger sample size are needed to increase the strength of the evidence for allograft usage in immunocompromised children. Last but not least, our case demonstrates that a high index of suspicion, prompt surgical intervention, and close multidisciplinary coordination between surgery, paediatrics, oncology, and plastic and reconstructive teams can lead to optimal outcomes in this vulnerable group of patients.

## Patient perspective

The patient was followed up closely post-operatively in the outpatient clinic, and the patient recovered well. The parents expressed their gratitude to the medical team for care they had received in hospital and during the daycare follow-up, which allowed the patient to undergo further treatment for his relapsed ALL.

## Data Availability

The original contributions presented in the study are included in the article/Supplementary Material, further inquiries can be directed to the corresponding author.
